# Evaluation of the value of the VI-RADS scoring system in assessing muscle infiltration by bladder cancer

**DOI:** 10.1186/s40644-020-00304-3

**Published:** 2020-04-06

**Authors:** Ziyong Wang, Yunyun Shang, Ting Luan, Yi Duan, Jiansong Wang, Haifeng Wang, Jingang Hao

**Affiliations:** 1 Department of Urology, the second affiliated Hospital of Kunming Medical University, No. 374, Dianmian avenue, Wuhua district, Kunming city, 650101 Yunnan province China; 2 Department of Radiology, the second affiliated Hospital of Kunming Medical University, No. 374, Dianmian avenue, Wuhua district, Kunming city, 650101 Yunnan province China

**Keywords:** Bladder cancer, Multiparameter magnetic resonance imaging, Reporting and data system, Scoring, Staging

## Abstract

**Background:**

The Vesical Imaging-Reporting and Data System (VI-RADS) was created in 2018, and a 5-point VI-RADS scoring system was proposed to determine whether the muscularis of the bladder has been infiltrated by tumor tissues.

**Purpose:**

To verify the accuracy of the VI-RADS scoring system in predicting muscle-invasive bladder cancer and to explore its value in clinical application.

**Materials and methods:**

A total of 220 patients with bladder cancer who underwent multiparameter magnetic resonance imaging from January 2017 to June 2019 were selected. Then, two radiologists with equivalent qualifications gave their diagnoses of bladder tumors on T2-weighted imaging, diffusion-weighted imaging and dynamic contrast enhanced imaging. Meanwhile, the bladder tumor was also scored on the basis of the VI-RADS system; for multifocal tumors, the highest tumor load was selected for scoring. Furthermore, the final pathological results of the patients were unknown during the imaging diagnosis and scoring. Next, the VI-RADS score was compared with the pathological results after surgery, and the ability of the VI-RADS score to assess the degree of muscularis infiltration was finally analyzed.

**Results:**

A total of 220 patients were included in our study, including 194 males and 26 females. Among them, the pathological results were 113 cases of muscle-invasive bladder cancer and 107 cases of non-muscle-invasive bladder cancer. The results showed that there was a positive correlation between the pathological results and VI-RADS score (r = 0.821, *P* < 0.05). The area under the receiver operating characteristic curve of the VI-RADS score was 0.960 (95% CI: 0.937, 0.983). When the VI-RADS score was above 3, the sensitivity, specificity and accuracy of predicting muscle-invasive bladder cancer were 82.3, 95.3 and 88.64%, respectively.

**Conclusion:**

The VI-RADS scoring system has good diagnostic value in predicting the degree of tumor invasion and can be used to guide clinical decision-making and management.

## Introduction

Bladder cancer ranks as the ninth most frequently diagnosed cancer worldwide [1]. It is estimated that the number of new cases of bladder cancer in the United States will reach approximately 80,470 in 2019, accounting for approximately 4.57% of all new cancers, and the number of deaths will account for 2.91% of all cancer-related deaths [2]. Approximately 70% of initial bladder cancers are non-muscle-invasive bladder cancers (NMIBCs), including Ta, T1 and Tis [3]. Generally, the treatment of NMIBC is mainly transurethral resection of the bladder tumor (TURBT), supplemented by bladder perfusion chemotherapy or immunotherapy. For muscle-invasive bladder cancer (MIBC) and high-risk NMIBC, the treatment methods include radical cystectomy (RC), adjuvant/neoadjuvant chemotherapy, radiotherapy and immunotherapy, etc. [4, 5] The treatment decision in patients with bladder cancer is mainly determined on the basis of distinguishing NMIBC from MIBC, so it is important to accurately assess whether the muscularis is infiltrated (T-staging) before treatment. At present, the stage of bladder cancer is mainly determined by the combination of imaging (including CT, MRI, etc.) and pathological examination (tumor samples are obtained by diagnostic transurethral resection, and a biopsy is performed), but thus far, these methods are not completely accurate [6–10], so we urgently need a more reliable tool to assess the clinical stage and guide clinical management.

The Vesical Imaging ⁃ Reporting and Data System (VI⁃RADS) was published in 2018, providing a new method to determine whether the muscularis of the bladder has been infiltrated by tumor tissues [11]. Based on multiparametric MRI, the system proposes a standardized reporting criterion for T2-weighted imaging (T2WI), diffusion-weighted imaging (DWI) and dynamic contrast enhanced (DCE) imaging sequences and establishes a 5-point VI-RADS scoring system. VI-RADS scores 1~5 are respectively defined as muscle invasion is highly unlikely, muscle invasion is unlikely to be present, the presence of muscle invasion is equivocal, muscle invasion is likely, and invasion of the muscle and beyond the bladder is very likely. Nevertheless, the scoring system has not been routinely used in clinical practice, and there are currently no large-scale confirmatory studies; consequently, our study aimed to verify the accuracy of the VI-RADS scoring system in predicting muscle-invasive bladder cancer and to investigate the clinical value of this tool.

## Materials and methods

### Patient population

This study was conducted by the radiology department and urology department of our hospital, and the study contents were approved by the hospital medical ethics committee. As this was a retrospective study, informed consent from patients was waived.

A total of 220 patients, including 194 males and 26 females aged 31~89 years, were enrolled in the urology department of the Second Affiliated Hospital of Kunming Medical University in China from January 2017 to June 2019. All patients were first diagnosed with bladder cancer and had received no previous treatment; they all underwent TURBT, RC or partial cystectomy within 2 weeks after receiving a multiparameter MRI examination. The postoperative specimens were examined pathologically. In addition, the surgical specimens from TURBT contained the muscularis of the bladder, and muscle infiltration was assessed pathologically. The inclusion criteria for this study were as follows: (1) Patients who were initially diagnosed with bladder cancer received no treatment or received only diagnostic transurethral resection. (2) The final pathological results were confirmed to indicate a malignant bladder tumor. (3) Surgery was performed within 2 weeks of the multiparameter MRI examination. The exclusion criteria were as follows: (1) a history of bladder cancer metastasis, recurrence, or other tumors; (2) failure to undergo surgery within a short time after examination; and (3) images that could not be accurately analyzed for reasons such as inadequate bladder filling and severe image artifacts.

### Multiparameter MRI examination

The examination equipment used for the 220 patients was 3.0 T MRI, and a multichannel phased array external surface coil was used to collect images with a high spatial resolution and signal-to-noise ratio. Moreover, no cystoscopy or indwelling catheterization was performed in any patients within 2 to 3 days before examination, and intestinal preparation and bladder volume preparation were performed 4 ~ 6 h and 1 ~ 2 h before examination, respectively.

Each patient underwent an ultrasonic examination before MRI to ensure that the bladder volume was approximately 300 ml, and real-time MRI images were also used to determine adequate bladder filling. Scan areas included the following: bladder, proximal urethra, and pelvic lymph nodes; prostate (male); uterus, ovaries, fallopian tubes, and vagina (female). Finally, the key image sequences, including T2WI, DWI and DCE, were obtained in the transverse plane, coronal plane and sagittal plane, respectively.

### Multiparameter MRI image sequence score and VI-RADS score

**Fig. 1:**
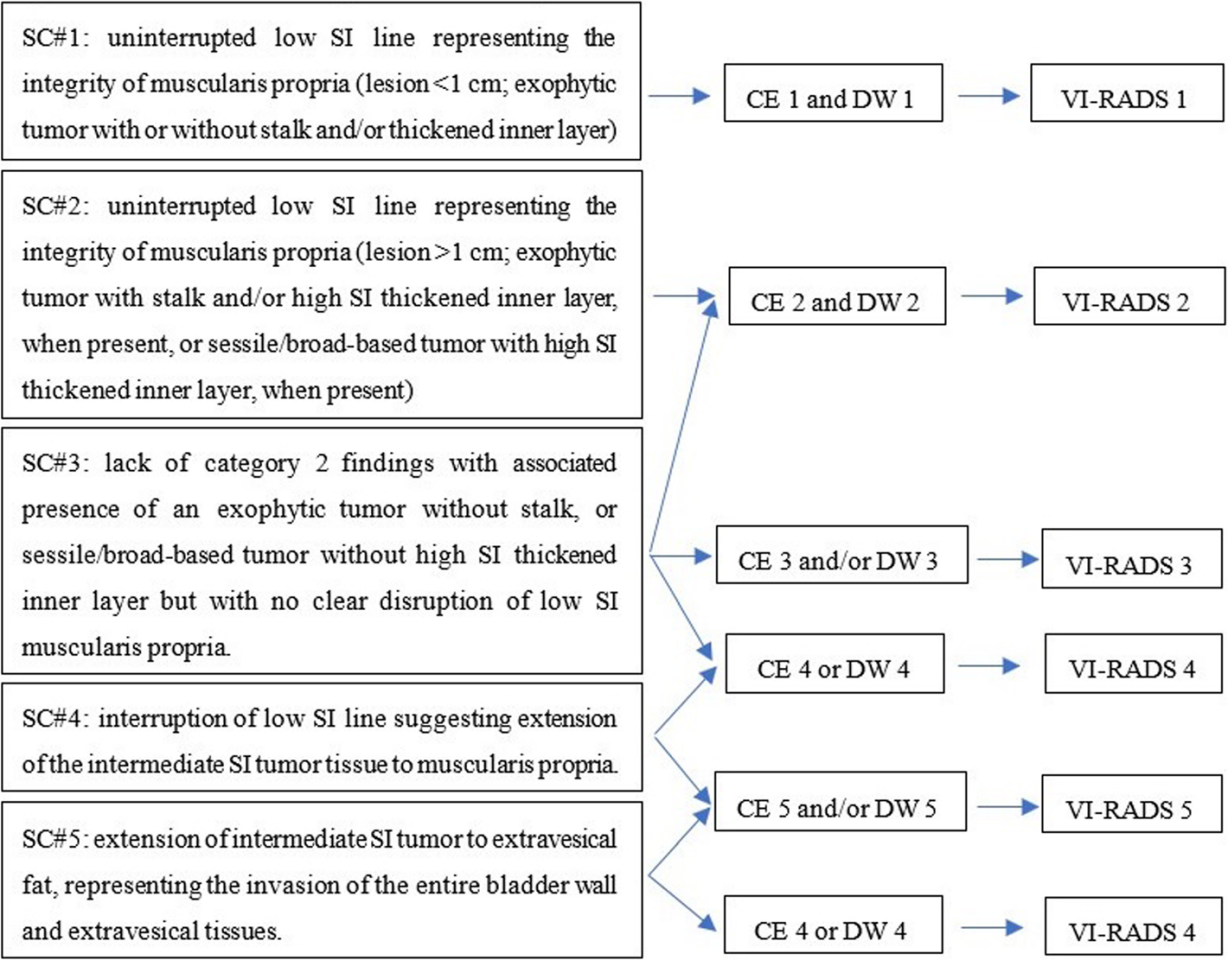
VI-RADS scoring rule schematic. CE = contrast enhanced; DW = diffusion weighted; SC = structural category; SI = signal intensity

The optimal plane of each image sequence was selected for analysis and diagnosis, and bladder tumors on T2WI, DWI and DCE images were scored on a 5-point scale according to the scoring criteria in the references [11] (Fig. 1). For multifocal tumors, we selected the one with the largest tumor load (the largest volume, the highest stage or the deepest depth of invasion) for the VI-RADS score. All imaging scores were completed independently by 2 radiologists with the same qualification (associate professors) at our hospital; when the results between the two readers were not consistent, the disagreement was resolved by consensus. It should be emphasized that the radiologists did not know the final pathological results of the patients when they scored the images.

### Statistical analysis

Statistical analysis was performed using SPSS version 19. The samples were divided into a non-muscle invasive group and a muscle invasive group. A chi-square test was used to compare the frequency distribution of the VI-RADS score, T2WI score, DCE score and DWI score of patients in the two groups, and an independent sample T-test was used to compare the mean differences of the scores between the two groups. We then used a linear correlation analysis to describe the relationship between the pathological stage and each score. Finally, we used receiver operating characteristic (ROC) curve analysis and the area under the ROC curve (AUC) to assess the diagnostic performance of the VI-RADS score, and we also evaluated the predictive ability of the VI-RADS score for MIBC by calculating the sensitivity, specificity and accuracy. The gold standard for diagnosis was the pathological result of the tumor, and all tests were bilateral; *p* < 0.05 was considered statistically significant.

## Results

A total of 220 patients were included in this study, including 194 males (88.18%) and 26 females (11.82%). The average age of the patients was 65.67 years (range: 31.0 ~ 89.0 years); for men, the average age was 65.23 years (range: 31.0 ~ 89.0 years); and for women, it was 68.96 years (range: 45.0 ~ 87.0 years). There were 113 cases (51.36%) and 107 cases (48.64%) in the muscle invasive group and non-muscle invasive group, respectively, and the final pathological results of 220 patients were as follows: Ta, 3 (2.80%); T1, 104 (97.20%); T2, 56 (49.56%); T3, 53 (46.90%); and T4, 4 (3.54%). Among all patients, TURBT, partial cystectomy and RC were performed in 56 cases (25.46%), 3 cases (1.36%) and 161 cases (73.18%), respectively.

Of the 220 patients, 25 patients (11.36%) were scored as VI-RADS 1, and all of them were divided into 0 groups. Sixty-one cases (27.73%) were classified as VI-RADS 2, including 58 cases (95.08%) in the non-muscle invasive group and 3 cases (4.92%) in the muscle invasive group. Thirty-six cases (16.36%) were classified as VI-RADS 3, including 19 cases (52.78%) in the non-muscle invasive group and 17 cases (47.22%) in the muscle invasive group. Thirty-six cases (16.36%) were classified as VI-RADS 4, including 5 cases (13.89%) in the non-muscle invasive group and 31 cases (86.11%) in the muscle invasive group. In addition, 62 cases (28.18%) were scored as VI-RADS 5 and were categorized into the muscle invasive group. The image sequence scores, VI-RADS scores and specific stages of the patients are shown in Table 1.

**Table 1:** Pathological stages corresponding to each image sequence score and VI-RADS scores in MIBC and NMIBC

Image sequences and scores	Non-muscle invasive group			Muscle invasive group			
	n	Ta	T1	n	T2	T3	T4
T2WI							
1	25	3	22	0	0	0	0
2	42	0	42	3	3	0	0
3	37	0	37	23	23	0	0
4	3	0	3	21	20	1	0
5	0	0	0	66	10	52	4
DCE							
1	25	3	22	0	0	0	0
2	58	0	58	3	3	0	0
3	19	0	19	18	18	0	0
4	5	0	5	31	31	0	0
5	0	0	0	61	4	53	4
DWI							
1	25	3	22	0	0	0	0
2	60	0	60	3	3	0	0
3	17	0	17	18	18	0	0
4	5	0	5	30	30	0	0
5	0	0	0	62	5	53	4
VI-RADS							
1	25	3	22	0	0	0	0
2	58	0	58	3	3	0	0
3	19	0	19	17	17	0	0
4	5	0	5	31	31	0	0
5	0	0	0	62	5	53	4

The mean VI-RADS scores for patients in the non-muscle invasive group and muscle invasive group were 2.04 and 4.35, respectively, and the difference was statistically significant (t = − 21.284, *P* < 0.05). The distribution of the VI-RADS score was significantly different between the two groups (x^2^ = 155.431, P < 0.05). The VI-RADS score of the non-muscle invasive group was mainly 1 ~ 3, while that of the muscle invasive group was mainly 3 ~ 5; additionally, the proportion of muscle infiltration to non-muscle infiltration was similar in patients with a score of 3. The mean VI-RADS score of all 220 patients was 3.22, and there was a positive correlation between the pathological results and VI-RADS score (r = 0.821, *P* < 0.05) in that the higher the VI-RADS score was, the greater was the possibility of muscle infiltration. The pathological results were also positively correlated with T2WI, DWI and DCE image scores, and the Pearson correlation coefficients were 0.785, 0.822 and 0.818, respectively. In particular, the correlation coefficient of the DWI image scores was the highest and was higher than the VI-RADS score.

**Fig. 2:**
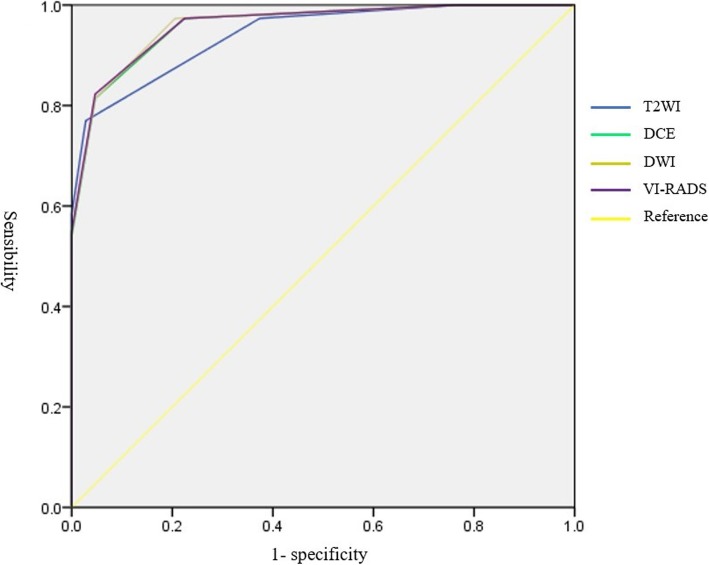
ROC curve for each image sequence score and VI-RADS score

Figure 2 and Table 2 respectively show the ROC curve and AUC of the VI-RADS score. The AUC of the VI-RADS score was 0.960 (95% CI 0.937, 0.983), while the AUC of the T2WI score, DCE score and DWI score were 0.941 (95% CI 0.913, 0.969), 0.959 (95% CI 0.936, 0.982) and 0.961 (95% CI 0.938, 0.983), respectively. As with the scores of each image sequence, the optimal critical value of the VI-RADS score for predicting muscle-invasive tumors was 3.5. The sensitivity and specificity of a VI-RADS score above 3.5 (that is, scores 4 and 5) for predicting muscle invasion were 82.3 and 95.3%, respectively, while the T2WI score was 77.0 and 97.2%, respectively, and the DCE score and DWI score were the same (81.4 and 95.3%, respectively). When the critical value was adjusted to 2.5, the sensitivity of the T2WI score, DCE score, DWI score and VI-RADS score was 97.3%, while the specificity was 62.6, 79.4, 77.6 and 77.6%, respectively. The sensitivity and specificity of each image sequence score and VI-RADS score under different critical values are shown in Table 3.

**Table 2:** The area under the ROC curve of each image sequence score and VI-RADS score

Variate	Area	Standard error	95% confidence intervals
T2WI	0.941	0.014	0.913, 0.969
DCE	0.959	0.012	0.936, 0.982
DWI	0.961	0.011	0.938, 0.983
VIRADS	0.960	0.012	0.937, 0.983

**Table 3:** Sensitivity and specificity of image sequence scores under different critical values

Variate	Critical value	Sensibility	1- specificity
T2WI	2.50	0.973	0.374
	3.50	0.770	0.028
DCE	2.50	0.973	0.224
	3.50	0.814	0.047
DWI	2.50	0.973	0.206
	3.50	0.814	0.047
VIRADS	2.50	0.973	0.224
	3.50	0.823	0.047

## Discussion

Accurate clinical staging not only can improve the clinical management of patients with bladder cancer but also can guide surgeons to devise the best surgical plan, and patients can also obtain a better prognosis [12, 13]. However, clinical staging is often difficult to accurately evaluate, which could lead to undertreatment or overtreatment. Multiparameter MRI has been proven to be highly accurate in assessing muscle invasion of bladder cancer [14], but how to comprehensively evaluate each image sequence and formulate unified VI-RADS scoring rules was not proposed until 2018. We further evaluated the accuracy of the VI⁃RADS score from the perspective of imaging and surgery and explored the guiding value of the scoring system for clinical practice.

**Fig. 3:**
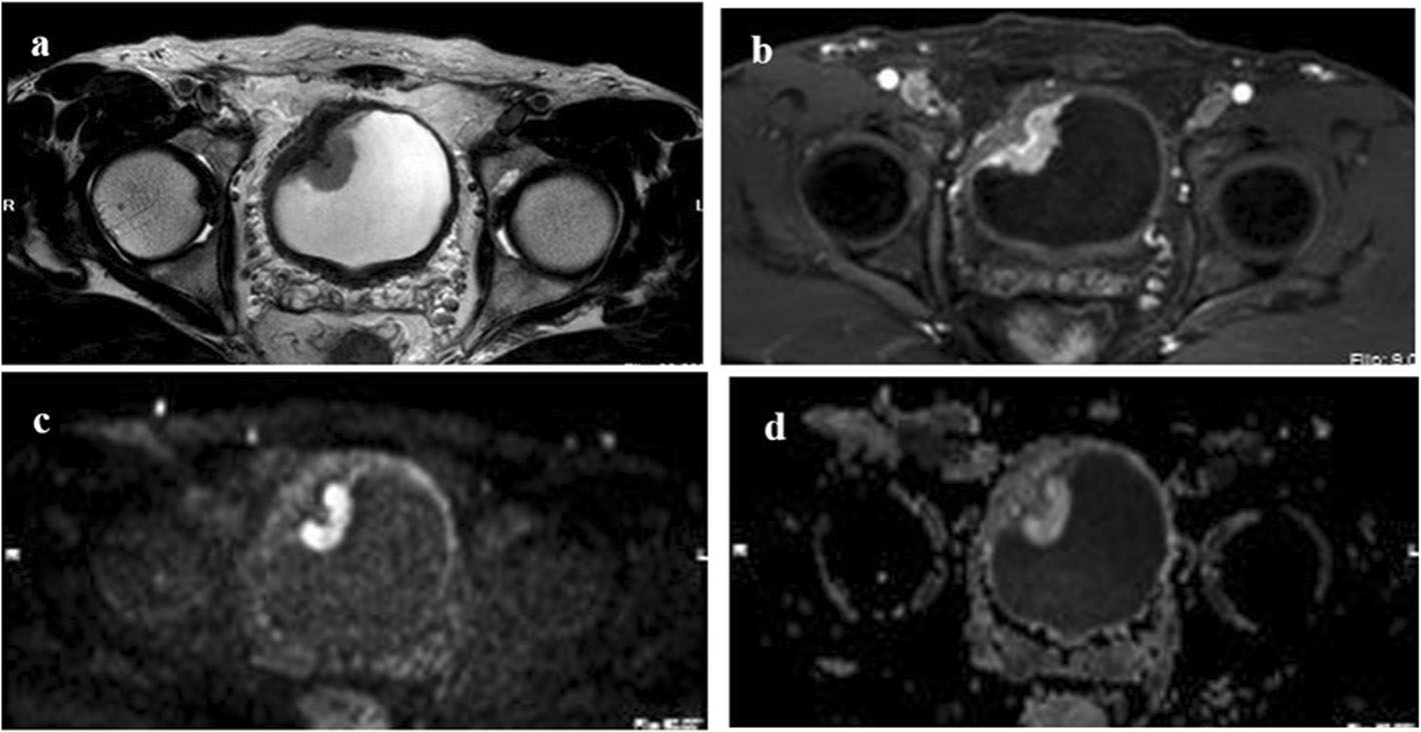
Image sequences of a single tumor with a score of VI-RADS 2 on the transverse plane. (**a**): T2-weighted imaging of tumors with a score of VI-RADS 2 on transverse plane; (**b**): Dynamic contrast enhanced imaging on transverse plane; (**c**)and(**d**): diffusion-weighted imaging on transverse plane

In our study, the VI-RADS score with a critical value of 3.5 had the best sensitivity (82.3%) and specificity (95.3%), which is similar to previous studies [15, 16]. This indicates that when the VI-RADS score is 4 or 5 points, MIBC can be assessed more reliably. The pathological results of all patients with a VI-RADS score of 1 were non-muscle infiltration, while cases with a VI-RADS score of 5 all involved muscle infiltration. The accuracy of assessing the degree of tumor muscle invasion was 100%, which provided strong reference evidence for the choice of surgical methods. The difference was that non-muscle invasion and muscle invasion accounted for 52.78 and 47.22% of the tumors with VI-RADS 3, respectively, indicating that the degree of tumor invasion in this portion of the patients was difficult to assess and the surgeons thus needed to think more carefully about which surgical scheme to choose. In addition, non-muscle invasion and muscle invasion accounted for 95.08 and 4.92% of the tumors with VI-RADS 2 scores and 13.89 and 86.11% of the tumors with VI-RADS 4 scores, respectively. The results may also show that VI-RADS scores of 2 and 4 still have high accuracy in assessing tumor muscle invasion, which can be of great help to surgeons in surgical decision-making. For instance, for a single tumor with a score of 2, even if the tumor is large or has a broad base, the surgeon still has the confidence to conduct TURBT directly. Nevertheless, we found that a single tumor with a score of VI-RADS 2 subjected to RC, and the pathological stage was eventually confirmed to be non-muscle infiltration (MRI images are shown in Fig. 3). We believe that there is overtreatment in these patients and that if clinicians use the VI-RADS score as a reference, they may develop a better and more reasonable surgical plan for patients.

The diagnostic performance of each image sequence in our study was also compared. The best critical values of the T2WI score, DWI score and DCE score in assessing muscle invasive tumors were 3.5, but the sensitivity of T2WI was the worst (77.0%), while that of DCE and DWI was 81.4%. Furthermore, the areas under the ROC curve of the three image sequences were 0.941, 0.959 and 0.961, respectively, indicating that T2WI and DWI have the worst and optimal diagnostic performance, respectively, which is consistent with the previous research reports [17]; DWI images have higher accuracy for T staging of bladder cancer, while there tends to be a higher incidence of excessive staging with T2WI and DCE [18, 19]. Therefore, as stated in the scoring rules [11], our results support that the dominant sequence for risk estimates is as follows: DWI (first) and DCE (second; especially if the DWI is suboptimal), and if there is any discordance between T2WI and DCE, such as a deviation of two categories, it may be more representative to choose DWI as the main score, especially when the image quality of the DWI sequence is optimal [11].

The pathological results are regarded as the gold standard to evaluate the accuracy of the VI-RADS score, but the accuracy of pathological results still needs to be considered. Whether there is muscle tissue in the specimen is very important for tumor staging, especially for tumors treated with TURBT, because the loss of muscle will lead to the T2 stage being mis- or underestimated as the Ta or T1 stage [20]. For tumors at the T1 stage after the first TURBT, up to 20% are modified .to the T2 stage after secondary TURBT, and even worse, fewer than 50% of the specimens in the first operation contain muscle tissue [21], so it is not absolutely reliable to evaluate the accuracy of VI-RADS in patients who have undergone TURBT. Pathologists can intuitively assess the location and size of the tumors in the specimens after RC compared with the surgical specimens of TURBT, and the materials used to make pathological sections include the full layer of the bladder, so the assessment of tumor stage is more accurate. Therefore, it may be more reliable to evaluate the VI-RADS in the tumors treated with RC, which needs to be verified by more prospective studies in the future. It is possible that in the presence of multiple lesions, pathologists may omit tumors with higher pathological stages when making sections; therefore, we believe pathologists may reduce missed diagnoses if they refer to MRI images and results.

**Fig. 4:**
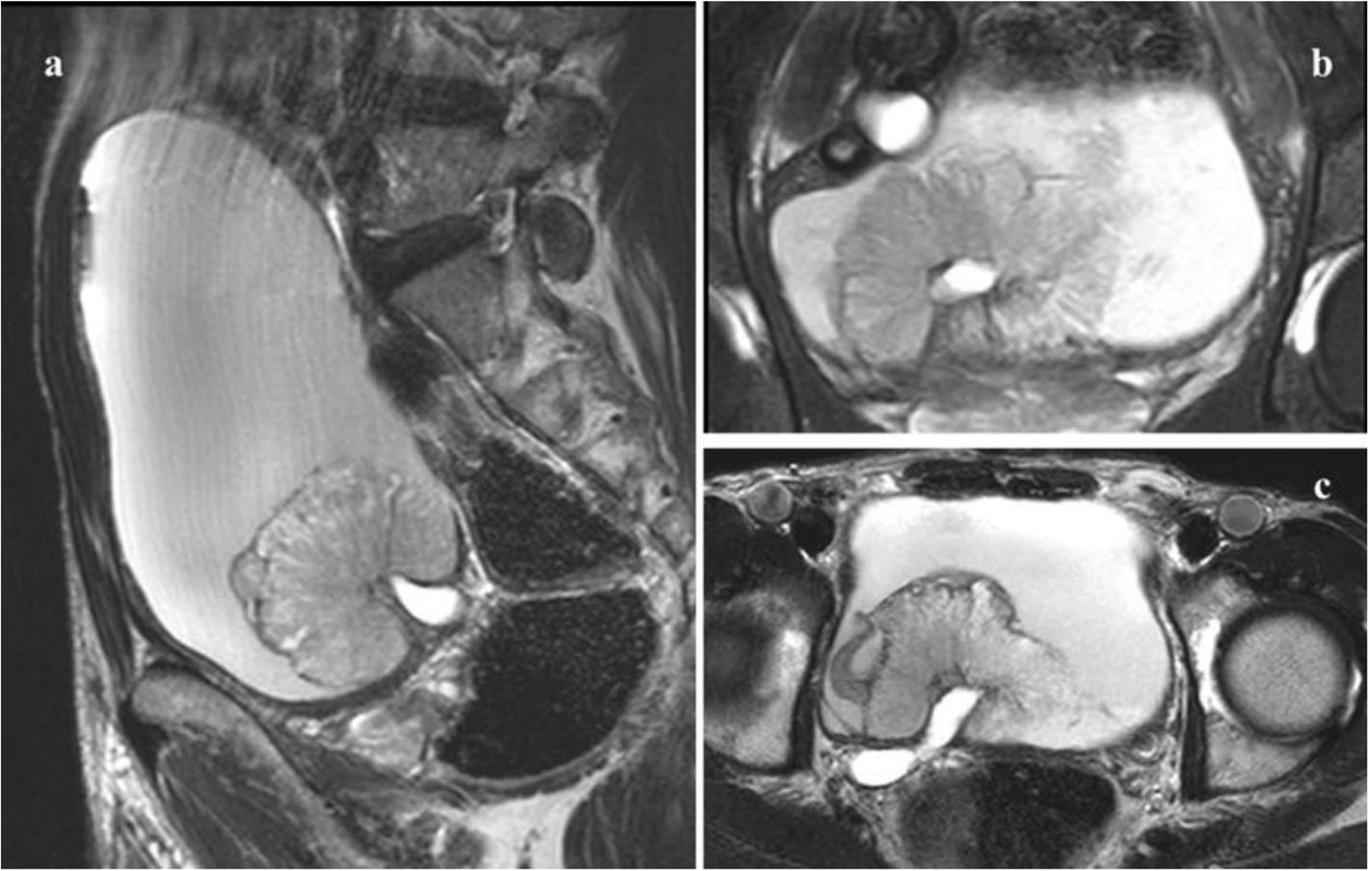
T2WI images of bladder tumors located around the ureter orifice on all scanning planes. **a** T2-weighted imaging of bladder tumor around the ureter orifice on the sagittal plane; **b** T2-weighted imaging on the coronal plane; **c** T2-weighted imaging on the transverse plane

In our study, the image diagnosis and final VI-RADS score were completed by consensus among 2 radiologists to reduce the bias caused by human factors. In the scoring process, the plane with the clearest image and clear relationship between tumor and muscularis was selected. However, approximately one-third of new tumors arise from the trigone, bladder neck, and ureteral orifice region [22]. The imaging diagnosis of these sites is difficult, and there may be differences in scores on different planes. The optimal plane (the plane where the most accurate score can be obtained) is not easily determined, and the scoring rules are not detailed. Figure 4 shows the tumor image characteristics of these sites. Therefore, we put forward another hypothesis about the existing scoring rules. For tumors at different sites, we can obtain VI-RADS scores on the sagittal plane, coronal plane and transverse plane, respectively, according to the scoring rules, and then verify the accuracy of different VI-RADS scores to determine which plane is optimal. This may be a further complement to the scoring rules. However, further studies are needed to confirm its feasibility.

Our study had some limitations. First, because of its retrospective nature, there may be a strong selection bias. Second, the VI-RADS score was decided by 2 radiologists after reaching an agreement, but the consistency among the evaluators was not examined. Third, the best criterion for the VI-RADS score should be the pathological results of patients after RC, but some of the pathological results in our study come from TURBT, which may affect the accuracy of VI-RADS score. Finally, our study is a single-center study, and the research samples are still relatively small. All patients have the same conditions for MRI examination, and doctors in each department have a very similar work experience and technical level.

## Conclusion

In conclusion, our study results suggest that the VI-RADS scoring system has good sensitivity, specificity and accuracy in assessing muscle invasion of bladder cancer. The VI-RADS scoring system can be used to guide the clinical management of patients. However, it should be noted that more careful consideration should be given to tumors with a VI-RADS score of 3, as well as tumors that occur in the bladder triangle, ureter orifice and around the bladder neck orifice.

## Data Availability

Not applicable.

## Abbreviations

AUC: Areas under the receiver operating characteristic curve
DWI: Diffusion-weighted imaging
DCE: Dynamic contrast enhanced
MIBC: Muscle-invasive bladder cancer
NMIBC: Non-muscle-invasive bladder cancer
RC: Radical cystectomy
ROC: Receiver operating characteristic curve
TURBT: Transurethral resection of bladder tumor
T2WI: T2-weighted imaging
VI-RADS: Vesical imaging-reporting and data system

## References

[1] Antoni S, Ferlay J, Soerjomataram I (2017). Bladder Cancer incidence and mortality: a global overview and recent trends. Eur Urol.

[2] Siegel RL, Miller KD, Jemal A (2019). Cancer statistics, 2019. CA Cancer J Clin.

[3] Ro JY, Staerkel GA, Ayala AG (1992). Cytologic and histologic features of superficial bladder cancer. Urol Clin North Am.

[4] Chang SS, Bochner BH, Chou R (2017). Treatment of non-metastatic muscle-invasive bladder Cancer: AUA/ASCO/ASTRO/SUO guideline. J Urol.

[5] Alfred Witjes J, Lebret T, Comperat EM (2017). Updated 2016 EAU guidelines on muscle-invasive and metastatic bladder Cancer. Eur Urol.

[6] Martingano P, Stacul F, Cavallaro M (2010). 64-slice CT urography: 30 months of clinical experience. Radiol Med.

[7] Paik ML, Scolieri MJ, Brown SL (2000). Limitations of computerized tomography in staging invasive bladder cancer before radical cystectomy. J Urol.

[8] Lin WC, Chen JH (2015). Pitfalls and limitations of diffusion-weighted magnetic resonance imaging in the diagnosis of urinary bladder Cancer. Transl Oncol.

[9] Tekes A, Kamel I, Imam K (2005). Dynamic MRI of bladder cancer: evaluation of staging accuracy. AJR Am J Roentgenol.

[10] Ark JT, Keegan KA, Barocas DA (2014). Incidence and predictors of understaging in patients with clinical T1 urothelial carcinoma undergoing radical cystectomy. BJU Int.

[11] Panebianco V, Narumi Y, Altun E (2018). Multiparametric magnetic resonance imaging for bladder Cancer: development of VI-RADS (Vesical imaging-reporting and data system). Eur Urol.

[12] Gore JL, Lai J, Setodji CM (2009). Mortality increases when radical cystectomy is delayed more than 12 weeks: results from a surveillance, epidemiology, and end results-Medicare analysis. Cancer.

[13] Shariat SF, Palapattu GS, Karakiewicz PI (2007). Discrepancy between clinical and pathologic stage: impact on prognosis after radical cystectomy. Eur Urol.

[14] Woo S, Suh CH, Kim SY (2017). Diagnostic performance of MRI for prediction of muscle-invasiveness of bladder cancer: a systematic review and meta-analysis. Eur J Radiol.

[15] Barchetti G, Simone G, Ceravolo I, et al. Multiparametric MRI of the bladder: inter-observer agreement and accuracy with the Vesical Imaging-Reporting and Data System (VI-RADS) at a single reference center. Eur Radiol. 2019;29(10):5498–506. 10.1007/s00330-019-06117-8 30887202

[16] Wang H, Luo C, Zhang F (2019). Multiparametric MRI for bladder Cancer: validation of VI-RADS for the detection of detrusor muscle invasion. Radiology.

[17] Huang L, Kong Q, Liu Z (2018). The diagnostic value of MR imaging in differentiating T staging of bladder Cancer: a meta-analysis. Radiology.

[18] Takeuchi M, Sasaki S, Ito M (2009). Urinary bladder cancer: diffusion-weighted MR imaging--accuracy for diagnosing T stage and estimating histologic grade. Radiology.

[19] Watanabe H, Kanematsu M, Kondo H (2009). Preoperative T staging of urinary bladder cancer: does diffusion-weighted MRI have supplementary value?. AJR Am J Roentgenol.

[20] Babjuk M, Bohle A, Burger M (2017). EAU guidelines on non-muscle-invasive Urothelial carcinoma of the bladder: update 2016. Eur Urol.

[21] Dalbagni G, Vora K, Kaag M (2009). Clinical outcome in a contemporary series of restaged patients with clinical T1 bladder cancer. Eur Urol.

[22] Paner GP, Montironi R, Amin MB (2017). Challenges in pathologic staging of bladder Cancer: proposals for fresh approaches of assessing pathologic stage in light of recent studies and observations pertaining to bladder Histoanatomic variances. Adv Anat Pathol.

